# Sero-prevalence of latent *Toxoplasma gondii *infection among HIV-infected and HIV-uninfected people in Addis Ababa, Ethiopia: A comparative cross-sectional study

**DOI:** 10.1186/1756-0500-2-213

**Published:** 2009-10-23

**Authors:** Techalew Shimelis, Mekashaw Tebeje, Endale Tadesse, Belete Tegbaru, Ashenafi Terefe

**Affiliations:** 1 Hawassa University, Department of Medical Laboratory Science, Hawassa, Ethiopia; 2 Ethiopian Health and Nutrition Research Institute, National HIV Laboratory, Addis Ababa, Ethiopia; 3 Jimma University, School of Medical Laboratory Technology, Jimma, Ethiopia

## Abstract

**Background:**

Toxoplasmosis in immuno-compromised hosts manifests primarily as a life threatening condition, toxoplasmic encephalitis. However, there is scarce information about the magnitude of *Toxoplasma gondii *infection among HIV-infected people in Ethiopia. This study was, therefore, conducted to determine the sero-prevalence of *T. gondii *infection among HIV-infected and HIV-uninfected subjects.

**Findings:**

Sera were collected from people with and without HIV infection for the purpose of studying hepatitis B virus (HBV) at St. Paul Hospital, Addis Ababa, Ethiopia from 24 January 2007 to 15 February 2007. Among these sera, the first 330 consecutive sera, 165 from each HIV sero-group, were selected and tested for anti-*T. gondii *IgG antibodies using Enzyme Linked Immunosorbent Assay. The seroprevalence of *Toxoplasma *infection was assessed against socio-demographic characteristics, HIV and HBV serostatus and HBV-related risk factors. The overall sero-prevalence of latent *T. gondii *infection among the study subjects was 90.0%. *Toxoplasma *infection was observed with respective prevalence of 93.3% and 86.7% among HIV-infected and HIV-uninfected people. Though *Toxoplasma *infection seems to be influenced by age, gender and HIV serostatus, only HBV serostatus was significantly associated (OR 2.71, CI 1.12 to 6.57) in multivariate logistic regression analysis.

**Conclusion:**

The seroprevalence of latent *T. gondii *infection is high and similar by HIV status. Educating people to prevent acquisition of new *Toxoplasma *infection and minimizing the risk of disease manifestations among HIV-*Toxoplasma *co-infected individuals is important.

## Background

*Toxoplasma gondii *is one of the most prevalent protozoan parasites of man and livestock [1]. It has been estimated that up to one third of the world's population is infected by *T. gondii *[2]. Most infections among humans occur by eating undercooked or raw meat containing tissue cysts or by exposure to oocysts through ingestion of contaminated foods and drinks with cat's faeces [3-5]. Other modes of transmission include the transplacental route, blood product transfusion and tissue transplantation [6,7].

In vast majority of immunocompetent human host, *T. gondii *ensue a latent infection characterized by the persistence of the organism in tissues (primarily brain, skeletal muscle, and heart) without causing disease [8]. However, in chronically infected individuals who develop defects in cell-mediated immunity a symptomatic disease more likely occurs as a result of reactivation of latent infection [9,10]. Toxoplasmosis among Acquired Immunodeficiency Syndrome (AIDS) patients manifests primarily as a life threatening condition, toxoplasmic encephalitis (TE) [9-11]. Early diagnosis and appropriate management of toxoplasmosis decreases the incidence rates of TE; subsequently reduce morbidity and mortality among HIV infected individuals [7].

In Ethiopia, up to 80% prevalence of *Toxoplasma *infection has been reported in different risk groups [12-15]. Although latent *Toxoplasma *infection has great importance among HIV infected people, it has been poorly studied. Therefore, this study was conducted to determine the sero-prevalence of latent *T. gondii *infection among HIV-infected and HIV-uninfected subjects in order to get some baseline information, from which clinical implication may be drawn.

## Methods

From 24 January 2007 to 15 February 2007, blood samples were collected from a total of 305 HIV-positive and 315 HIV-negative clients seeking either HIV or immunological testing at St. Paul's Hospital, Addis Ababa, Ethiopia. Parts of separated sera were originally used for studying seroprevalence of hepatitis B virus (HBV) infection and leftover samples were stored at -70°C for further investigations. Detailed description of methods used to study HBV infection was published elsewhere [16].

HIV and HBV sero-status of all samples were, therefore, known and selected sera from each HIV serogroup were used for the purpose of studying seroprevalence of *Toxoplasma *infection. In the present study, because of limited regent kits, we only included the first 330 consecutive serum samples, 165 from each HIV sero-group.

Laboratory investigation of *Toxoplasma *infection was carried out with in six months after initial blood collection. Frozen sera were thawed at room temperature and those having unsuitable appearance in terms of turbidity and hemolysis were not included. Sera were tested in duplicate for anti-*Toxoplasma *IgG antibody using the Enzyme Linked Immunosorbent Assay (BioCheck, Inc, CA, USA). Positive and negative controls were included per each batch of test run to ensure kits were working properly and technical procedures were carried out correctly. As per the instruction of the manufacturer, the mean absorbance value of each sample was divided by the cut - off calibrator mean value to obtain a Toxo G Index. A sample was considered positive for anti-*Toxoplasma *IgG antibody whenever a Toxo G Index value is equal or greater than 1.0 (> 32 IU/ml).

*Toxoplasma *infection was assessed against socio-demographic characteristics, HIV and hepatitis B core antibody (anti-HBc) serostatus. HBV-related risk factors including history of blood transfusion, tattooing, blood letting, multiple sexual partners, and surgery was also analyzed if they have association with *Toxoplasma *infection.

Data entry and analysis was performed using SPSS Version-12 software. Summaries were presented in terms of mean, range and percentage as appropriate. Differences in proportions were evaluated by Pearson's Chi-square test. In cases in which more than 20% of the expected values were less than 5, Fisher's exact test was used in lieu of chi-square test. Bivariate and Multivariate logistic regression analyses were used to assess the crude and adjusted effect of HIV and other correlates on *Toxoplasma *infection. Odds ratio was used to measure the strength of association between the *Toxoplasma *infection and its correlates. A statistical test result was considered significant whenever a p-value was < 0.05.

Institutional ethical clearance and appropriate written informed consent was obtained from each participant to use surplus sera for the present study.

## Results

A total of 330 sera were included for studying sero-prevalence of latent *Toxoplasma *infection among people with and without HIV infection. Half of the samples (50%) were HIV positives and 44.8% (148/330) had anti-HBc marker. The mean age of HIV infected participants was 35 years (range 20-66 years; SD 9.3) compared with mean age of 25 years (range 17-64 years; SD 7.3) in HIV uninfected clients. The male to female ratio was 0.8:1 in HIV positive and 1:1 in HIV negative individuals.

The cumulative sero-prevalence of anti-*Toxoplasma *IgG antibody among the study subjects was 90.0% (297/330). In bivariate analysis, rate of *Toxoplasma *infection was significantly higher among males than females (93.6% versus 86.6%; p = 0.04). In addition, rate of *Toxoplasma *infection was shown to increase significantly with age (p = 0.003) (Table 1).

**Table 1:** Sero-prevalence of anti-*Toxoplasma *IgG antibody in relation to socio-demographic characteristics of the study subjects, Addis Ababa, 2007.

***Characteristics***	***Total (%)***	***Number (%) positive for anti*-Toxoplasma *IgG antibody***	***P-value***
Residence			
Rural	24 (7.3)	24 (100)	0.15
Urban	306 (92.7)	273 (89.2)	
Sex			
Male	156 (47.3)	146 (93.6)	0.04
Female	174 (52.7)	151 (86.8)	
Age (years)			
17-24	106 (32.1)	87 (82.1)	0.003
25-34	132 (40.0)	122 (92.4)	
≥ 35	92 (27.9)	88 (95.7)	
Marital status			
Married	112 (33.9)	105 (93.8)	0.07
Single	167 (50.6)	144 (86.2)	
Divorced/Widowed	51 (15.5)	48 (94.1)	
Religion			
Christian	286 (86.7)	254 (88.8)	0.10
Muslim	44 (13.3)	43 (97.7)	
Ethnicity			
Amhara	148 (44.8)	131 (88.5)	0.70
Oromo	94 (28.5)	84 (89.4)	
Gurage	55 (16.7)	51 (92.7)	
Other	33 (10.0)	31 (93.9)	
Occupation			
Gov't/private employee	82 (24.8)	75 (91.5)	0.17
Housewife/house maid	56 (17.0)	51 (91.1)	
Student	59 (17.9)	48 (81.4)	
Merchant	40 (12.1)	38 (95.0)	
No work	93 (28.2)	85 (91.4)	
Educational status Educational stat			
Illiterate	37 (11.2)	36 (97.3)	0.09
Primary school	117 (35.5)	108 (92.3)	
Secondary school and above	176 (53.3)	153 (86.9)	

The prevalence of latent *Toxoplasma *infection was 93.3% (154/165) among HIV positive and 86.7% (143/165) among HIV negative participants. This difference was also statistically significant in bivariate analysis (p = 0.04). Regardless of their HIV status, anti-*Toxoplasma *IgG antibody was more frequently detected in sera with anti-HBc antibody compared with those with no anti-HBc serological marker (95.3% verses 85.7%, p = 0.004). However, similar rate of anti-*Toxoplasma *IgG antibodies was detected in sera positive for both HBV and HIV compared to those with HBV alone (48.7% versus 46.2%, p > 0.05). Any of HBV-related risk factors analyzed to have association with *Toxoplasma *infection were not found to be significant.

In further analysis, after adjustment for those significantly associated variables using the multivariate logistic regression, age, gender and HIV status were not found to affect rate of *Toxoplasma *infection. However, sera with anti-HBc antibody were shown more likely to have anti-*Toxoplasma *IgG antibody (OR 2.71, CI 1.12 to 6.57) (Table 2).

**Table 2:** Multivariate analysis on seemingly significant predictors of *T. gondii *infection in bivariate analysis, Addis Ababa, 2007

**Characteristics**	**Crude odds ratio** **(95% confidence interval)**	**Adjusted odds ratio (95% confidence interval)**	**P-value**
Sex			
Female	1	1	
Male	2.22 (1.02-4.83)	1.9 (0.84-4.32)	0.13
Age (years)			
17-24	1	1	
25-34	2.66(1.18-6.01)	2.33 (0.93-5.81)	0.07
≥ 35	4.81(1.57-14.70)	2.96 (0.81-10.81)	0.10
HIV status			
Negative	1		
positive	2.15 (1.01-4.60)	1.32 (0.52-3.33)	0.56
Anti-HBc status			
Negative	1	1	
Positive	3.56(1.41-7.97)	2.71 (1.12-6.57)	0.03

## Discussion

The sero-prevalence of anti-*Toxoplasma *IgG antibody among HIV-infected and HIV-uninfected participants was determined in the present study. The overall prevalence rate of latent *T. gondii *infection was found to be 90%. The high prevalence of anti-*Toxoplasma *IgG antibody observed in this hospital based study was in agreement with the result of a previous survey carried out in the general population of Ethiopia (75%) [13]. Similar sero-prevalence of *T. gondii *infection (80%) was also reported in a study, which involved HIV-infected and HIV-uninfected people in Addis Ababa [15]. The high sero-prevalence of latent *Toxoplasma *infection among the study population seems reasonable as raw or insufficiently cooked meat prepared in a various favorite cultural food is consumed. In addition, cats are abundant to cause environmental contamination and the climate is favorable to favor survival of the parasite.

In this study, sero-prevalence of latent *T. gondii *infection was shown to increase with age though the difference was not statistically significant. Similarly, no association between prevalence of latent *Toxoplasma *infection and age was reported elsewhere [17]. In contrast, significantly higher risk of having latent *Toxoplasma *infection was observed with increasing age [18,19]. The non-significant effect of age on *T. gondii *infection in our study subjects may suggest that exposure was irrespective of the risk behaviors pertinent to specific age group.

Moreover, the present study showed that HIV-positive and HIV-negative subjects had similar exposure to *T. gondii *infection, as reported by others [15,17,18]. The high prevalence of latent *T. gondii *infection in these risk groups, particularly among HIV-infected people, is of great concern. Evidences have shown that high prevalence of latent *T. gondii *infection and level of immunodeficiency in a given population are directly related to the incidence of TE [7,20]. It has been estimated that approximately one-third of HIV positive people with latent *T. gondii *infection will develop toxoplasmosis [11]. Accordingly, high incidence rate of TE in our study population is expected unless preventive measures are undertaken.

However, our result indicating the more frequent occurrence of latent *T. gondii *infection among people with anti-HBc antibody needs further study, as a similar observation is not yet reported, in which additional results may confirm or challenge our finding. The lack of association between *Toxoplasma *serostatus and HBV-related risk-behaviors including blood transfusion may be the role of these routes in the transmission of *Toxoplasma *infection was non-significant among the study population.

Finally, because this study was not specifically designed to study toxoplasmosis, we missed data on basic risk factors such as cat ownership, dietary habits, soil exposure and other important risk factors for disease acquisition. Moreover, because of limited resources, sufficient number of sera was not included so that the study has lower statistical power. Our findings, therefore, should be interpreted in light of the study limitations.

In conclusion, the sero-prevalence of latent *T. gondii *infection was high and similar among HIV-infected and HIV-uninfected people. Therefore, burden of *T. gondii *infection should not be overlooked and educating people to prevent acquisition of new infection has great importance. Efforts should also be made to minimize the risk of subsequent reactivation and disease manifestation among HIV-positive people with latent *Toxoplasma *infection.

## Competing interests

The authors declare that they have no competing interests.

## Authors' contributions

TS, AT and BT designed the study protocol. TS and ET performed data analysis. MT carried out the laboratory work. All authors wrote the manuscript.
